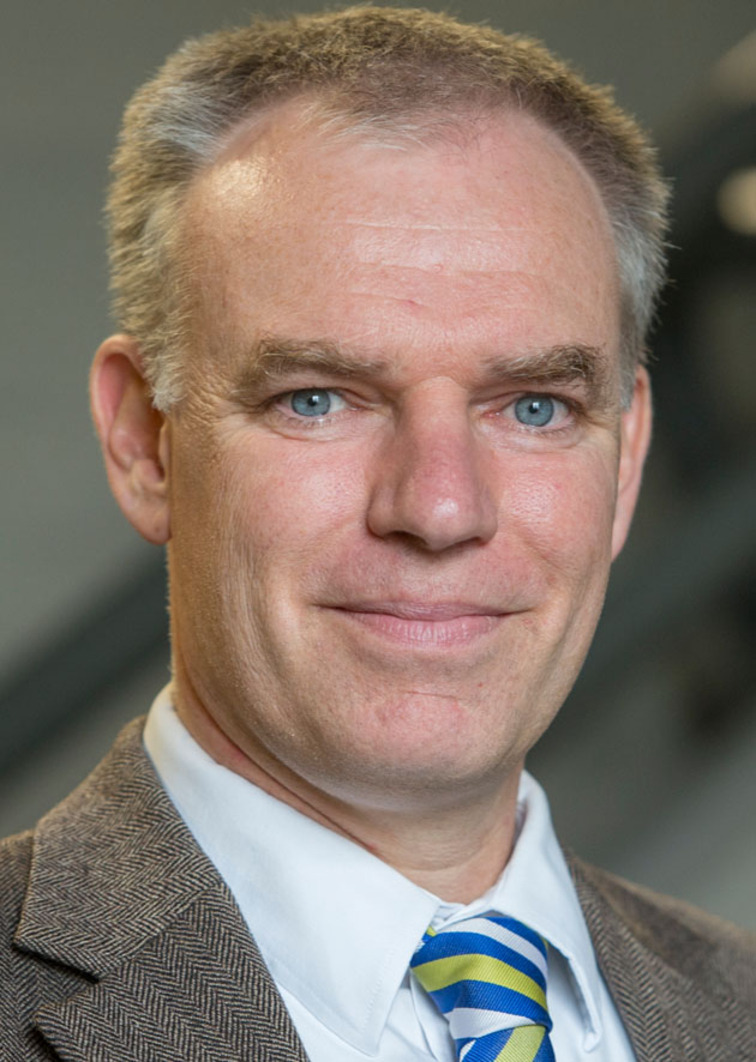# Charge Matters: Mutations in Omicron Variant Favor Binding to Cells

**DOI:** 10.1002/cbic.202100681

**Published:** 2022-02-02

**Authors:** Chuanxiong Nie, Anil Kumar Sahoo, Roland R. Netz, Andreas Herrmann, Matthias Ballauff, Rainer Haag

**Affiliations:** ^1^ Institut für Chemie und Biochemie Freie Universität Berlin Takustrasse 3 14195 Berlin Germany; ^2^ Fachbereich Physik Freie Universität Berlin 14195 Berlin Germany; ^3^ Max Planck Institute of Colloids and Interfaces 14476 Potsdam Germany

## Abstract

Evidence is strengthening to suggest that the novel SARS‐CoV‐2 mutant Omicron, with its more than 60 mutations, will spread and dominate worldwide. Although the mutations in the spike protein are known, the molecular basis for why the additional mutations in the spike protein that have not previously occurred account for Omicron's higher infection potential, is not understood. We propose, based on chemical rational and molecular dynamics simulations, that the elevated occurrence of positively charged amino acids in certain domains of the spike protein (Delta: +4; Omicron: +5 vs. wild type) increases binding to cellular polyanionic receptors, such as heparan sulfate due to multivalent charge‐charge interactions. This observation is a starting point for targeted drug development.

## Graphical abstract

**Because of the mutations** present in the Omicron variant, the surface of the spike protein is highly positively charged. For binding of SARS‐CoV‐2 to host cells, electrostatic interactions with heparan sulfate polyglycerols covering the cell surface play an essential role. Molecular dynamic simulation reveals that the interaction of the Omicron spike with polysulfates is much stronger than that of the wild type. We surmise that this enhanced interaction favors binding of Omicron and therefore subsequent cell entry and infection.

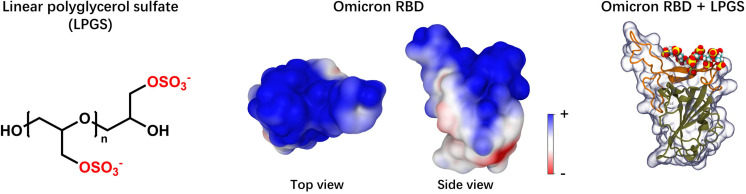

## Introduction

The severe acute respiratory syndrome coronavirus type 2 (SARS‐CoV‐2) has caused a pandemic of unprecedented severity for almost two years, with more than 200 million infections and 4 million deaths. Despite successful vaccination strategies its prevention and inhibition are still a challenge and, thus, the focus of current research. The virus mutates quickly, and several mutants have turned out to be more infectious than the wild type.^[1]^ In addition, these variants are of increased viral fitness and resistance towards vaccines compared to the wild type.^[2]^ In consequence, the variants have replaced the wild type worldwide.^[3]^ The Omicron variant being the latest mutant is of particular concern because based on the more than 60 mutations all present knowledge points potentially to an even enhanced infectivity. Hence, a better understanding of the interaction of SARS‐CoV‐2 with its respiratory host cells, especially the early stage of cellular uptake of the virion, is of central importance. Here, we will provide strong arguments that mutations of the spike protein of the Omicron variant are favorable for the very first step of cell infection ‐ the attachment to the cell surface.

### Electrostatics and Uptake of Virus

**Figure 1:**
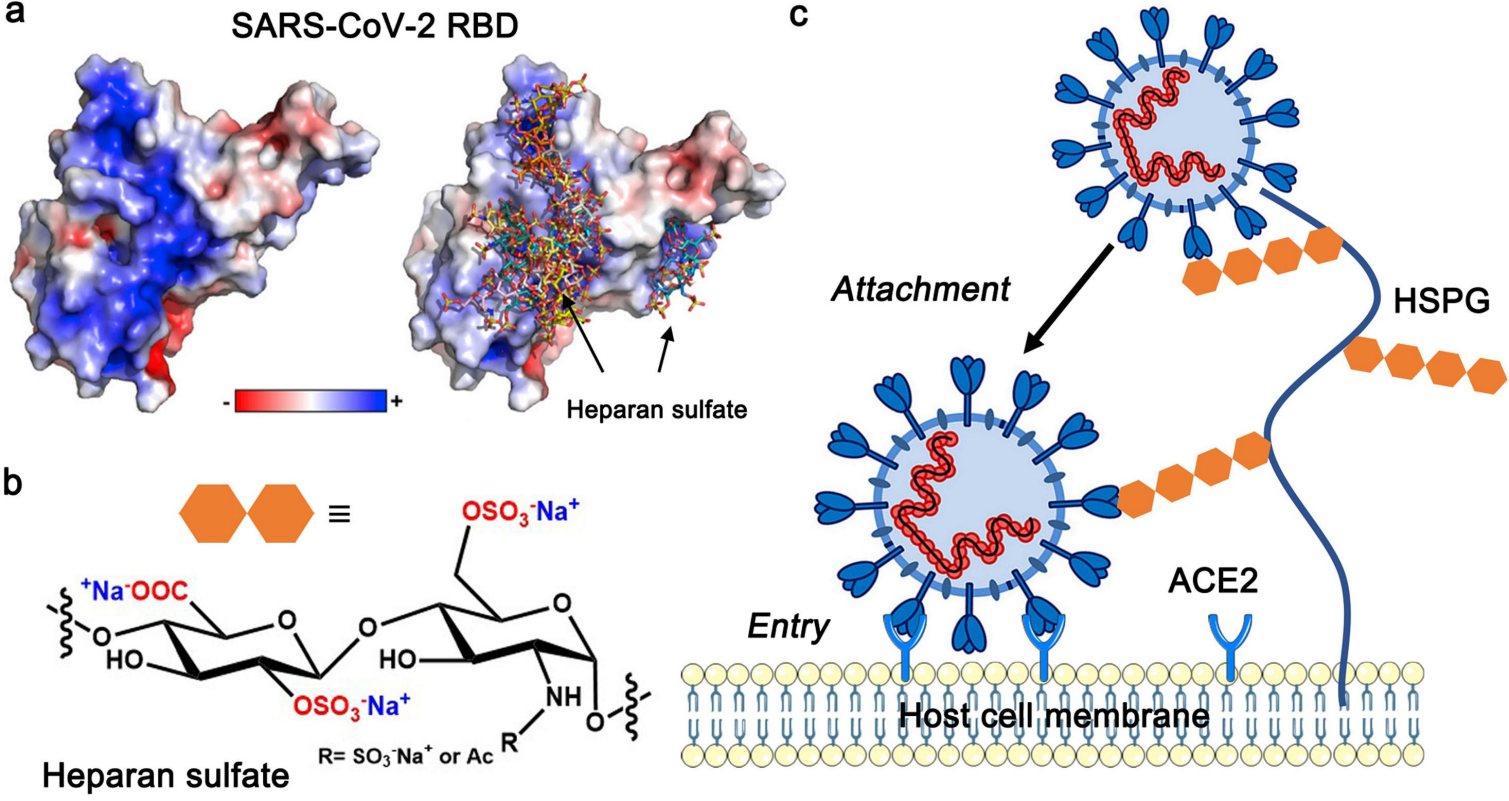
First steps of infection by SARS‐CoV‐2. a) Left: The electrostatic potential map of the RBD of wild type SARS‐CoV‐2 is represented. Right: Positively charged amino acids located on the surface of the homotrimeric organized spike protein (RBD: receptor binding domain) interact strongly with the highly negatively charged heparan sulfate moieties of the HSPG (only ectodomain of the spike protein is shown); b) repeating unit of heparan sulfate; c) early stages of cell infection by SARS‐CoV‐2. In the first step the spike proteins interact closely with heparan sulfate attached to the HSPG by strong electrostatic interaction. In a second step interaction with the ACE2‐receptor leads subsequently to the uptake of the virion into the cell. Reproduced with permissions from Ref. [4] and Ref. [5], respectively. Copyright 2020 Elsevier and 2021 Wiley‐VCH.

Initially, only the essential cellular ACE2 has been considered as a receptor for SARS‐CoV‐2 binding to the host cell. However, by now it is well‐established that the first and necessary step for uptake is the interaction of SARS‐CoV‐2 with the heparan sulfate proteoglycans (HSPG)^[6]^ on the cell surface.^[4]^ The HSPG consist of unbranched, negatively charged heparan sulfate (HS) attached to cell surface proteins. Figure 1 displays the structure of the HSPG together with the first two steps of virus infection: A core protein is linked covalently to linear chains of HS which is a highly sulfated glycosaminoglycan (GAG) with a degree of sulfation between 0.8 and 1.8 (degree of sulfation defined by the number of sulfated groups per repeating unit). The role of the HSPG for the attachment of a number of viruses has been discussed in detail by Cagno et al.^[6]^ who could establish clear evidence for the binding of various viruses to the HSPG. As an important conclusion, Cagno et al. suggested that the main reason for initial binding should be sought in an electrostatic interaction of the HS‐chains with positively charged patches on the surface of the viral proteins. In general, electrostatic interactions between highly charged GAGs and various proteins play a central role for many biological functions.^[7]^

The advent of SARS‐CoV‐2 has led to an enormous effort to elucidate the mechanism of cell entry of this virus. Very recent work has indeed underscored the importance of electrostatic interaction for the binding of SARS‐CoV‐2 to HS.^[8]^ The HS‐chains interact closely with a patch of positive charges located on the surface of the spike protein of SARS‐CoV‐2 as shown schematically in Figure 1. Chopping off the HSPG of cell surfaces by enzymes reduces the infection of SARS‐CoV‐2 significantly.^[4]^ Inhibition studies by highly charged polyelectrolytes provide another proof for the importance of charge‐charge interaction for binding.^[5]^ Thus, highly charged synthetic polyelectrolytes can compete with the HSPG and block the first step of virus infection as shown in Figure 1a. Hence, the first two steps of the process of virus uptake into cells can be envisioned as shown in Figure 1c: In step **1** the spike proteins on the surface of the virion are attached to the HSPG by strong charge‐charge interaction between the negatively charged polysulfate and the positively charged amino acids on the spike protein. In step **2**, the receptor binding domain (RBD) binds via its ‘up’ conformation to ACE2, essential for entry into the host cell.

Taken together, these investigations have clearly revealed that strong electrostatic interaction in step **1** is necessary for the uptake of the virion during cell infection. Here we discuss evidence for the central role of electrostatic interaction for the much higher transmissibility of the recent mutants of SARS‐CoV‐2 with a focus on the new Omicron variant. Analyzing the results of recent investigations on the mutations in the spike protein, we come to the conjecture that the Omicron variant has a high potential to enter the cell more efficiently than all other recent mutants.

### Overview of Mutations

**Figure 2:**
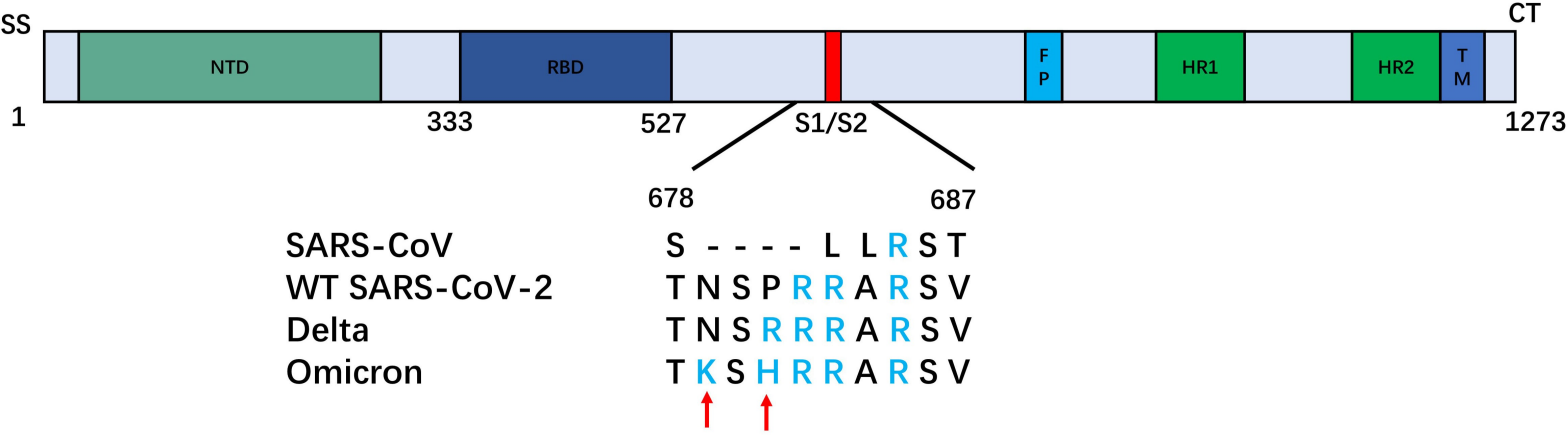
Survey of mutations at the S1/S2 domain of the spike protein. SS: signal sequence; NTD: N‐terminal domain; RBD: receptor‐binding domain; FP: fusion peptide; HR1: heptad repeat 1; HR2: heptad repeat 2; TM: transmembrane domain; CT: cytoplasmic tail; red: furin cleavage site. Positively charged amino acids are marked blue. Red arrows indicate two additional positively charged amino acids of the Omicron variant.

**Figure 3:**
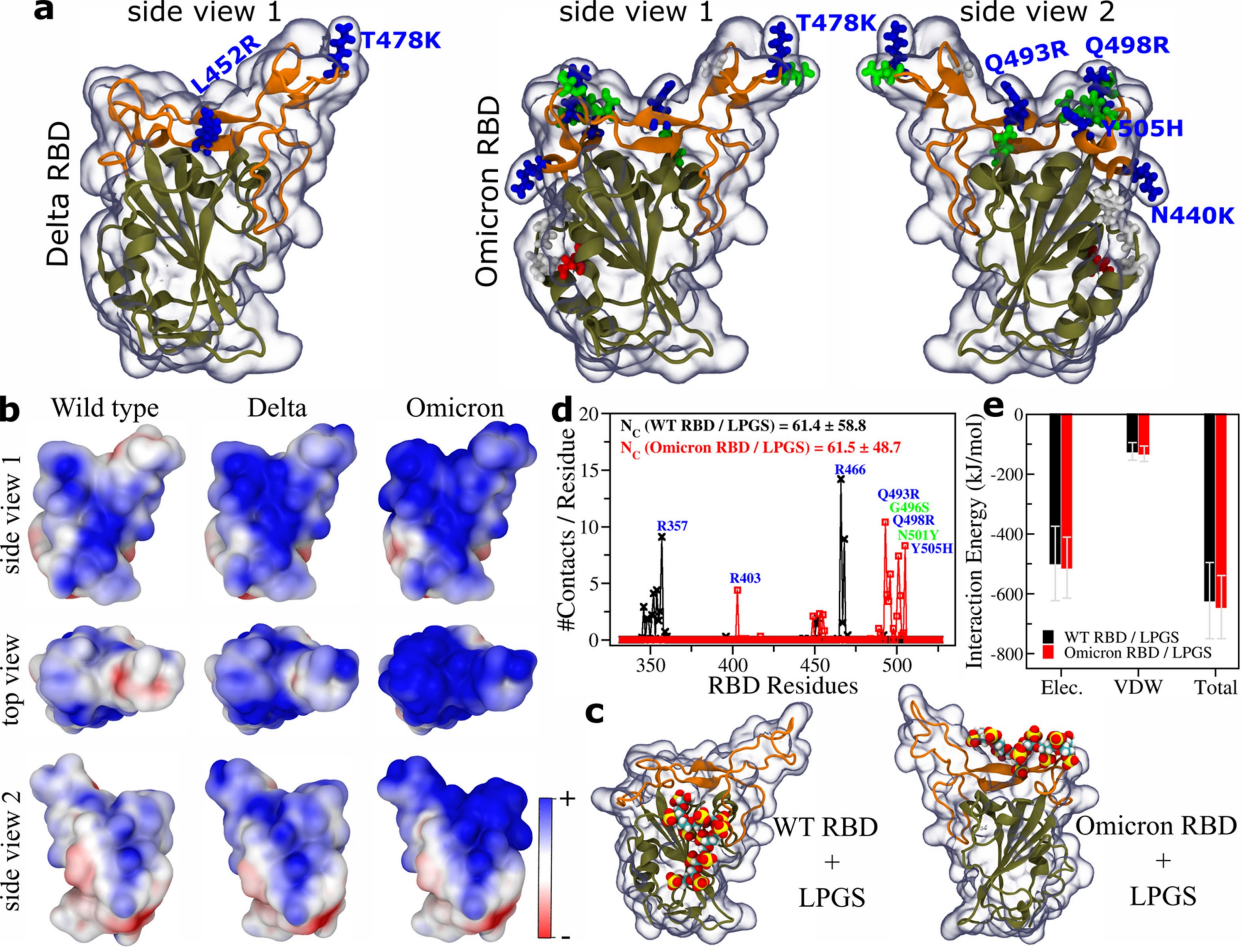
MD simulation results. a) Snapshots of the SARS‐CoV‐2 Delta and Omicron variant RBDs. The backbone atoms of the receptor binding motif are shown in orange, whereas the rest of the protein backbone is shown in tan. The mutated residues compared to the wild type, shown in ball‐stick representation, are colored according to the residue type: cationic (blue), anionic (red), polar (green), hydrophobic (white). Cationic residue names are also provided. b) Electrostatic potential maps for the wild type, Delta, and Omicron RBDs. c) Snapshots after 1000 ns and 900 ns of MD simulations, representing the complexation of a LPGS undecamer with the wild type and Omicron RBDs (for Omicron only LPGS bound to the new binding site is shown), respectively. LPGS is shown in the space filling representation: carbon (cyan), hydrogen (white), oxygen (red), and sulfur (yellow). d) The number of contacts LPGS forms with each RBD residue for the wild type and Omicron variant. e) Comparison of interaction energies of LPGS with the wild type and Omicron RBDs. The electrostatic (Elec.) and van der Waals (VDW) contributions to the total interaction energy are also provided. d and e) For Omicron only data for the new binding site are shown.

Figure 2 and Table 1 give an overview of the mutations in the RBD and the S1/S2‐domain of the Omicron variant. As a consequence of the mutations, the number of positively charged amino acids of the spike protein increased by 9 in Omicron compared with the wild type (wt) SARS‐CoV‐2, whereas in the case of the Delta variant there were only 4 more. More specifically, the S1/S2 domain exhibits a number of marked changes in which a negatively charged or neutral amino acid is replaced by a basic amino acid. Some of these new positive charges lead to an amplification of the Cardin‐Weintraub motif identified on the surface of the spike protein of wt SARS‐CoV‐2 by Kim et al.^[8b]^ The Cardin‐Weintraub^[9]^ corresponds to amino acid sequences of ‘XBBXBX’ and ‘XBBBXXBX’, where X is a hydrophobic amino acid and B denotes a basic residue, such as arginine, lysine and histidine. For a variety of proteins those sequences exposed on the protein surface have been shown to interact with the negatively charged sulfate groups present in GAGs.[^9^, ^10^] Thus, these positive patches can act as multivalent counterions of the GAG‐chains forming finally a stable complex (cf. the discussion in Ref. [7]). While the ectodomain of SARS‐CoV spike has only one basic amino acid in the S1/S2‐domain, Figure 2 reveals that this sequence turned into a surface exposed Cardian‐Weintraub motif ‘XBBXBX’ in wt‐SARS‐CoV‐2 spike^[9]^ and even extended along the mutations ongoing from the Delta mutant finally to the Omicron variant. In the Delta variant, this sequence has been enlarged considerably to the second type ‘XBBBXBX’ which must lead to an even stronger binding. Finally, the Omicron variant exhibits an even larger patch of positive amino acids (see below and Figure 3). The conservation and enrichment of such mutations provide a strong indication for a selective advantage very likely in initial binding to the host cell of respective virus variants.

**Table 1:** Summary of the spike mutations of Delta and Omicron variants compared to wild type.

Virus	Spike mutations	Number of mutations	Change of charge
B.1.617.2 (Delta variant)	T19R (↑), G142D (↓), Δ156, Δ157, R158G (↓), **L452R (↑), T478K (↑)**, D614G (↑), **P681R (↑)**, D950N (↑)	10	+4
B.1.1.529 (Omicron variant)	A67V, Δ69‐70, T95I, G142D (↓)/Δ143‐145, Δ211/L212I, ins214EPE (↓↓), **G339D (↓), S371L, S373P, S375F, K417N (↓), N440K (↑), G446S, S477N, T478K (↑), E484A (↑), Q493R (↑), G496S, Q498R (↑), N501Y, Y505H (↑)** , T547K (↑), D614G (↑), H655Y (↓), **N679K (↑), P681H (↑)**, N764K (↑), D796Y (↑), N856K (↑), Q954H (↑), N969 K (↑), L981F	32	+9

Notes. ↑: add one positive charge, ↓: add one negative charge. The mutations in RBD are highlighted, and the mutations at S1/S2 domain are underlined.

### MD Simulation for Omicron RBD Binding with Polysulfates

The electrostatic potential (ESP) map reveals that the wild type RBD has a cationic patch a certain distance away from the receptor binding motif (RBM) (see Figure 3b, side view 1). This cationic patch, because of the two charged mutations L452R and T478K, becomes larger for the Delta RBD. For the Omicron RBD, an additional cationic patch is produced by three neighboring charged mutations (Q493R, Q498R, and Y501H) which is located right on the RBM, see Figure 3b top view and side view 2. The Omicron RBD, because of this additional cationic patch, is expected to bind strongly to negatively charged HSPGs present on the cell surface.

To test this, we consider here a simpler polysulfate compared to HS on the cell surface, namely a linear polyglycerol sulfate (LPGS) undecamer and compare its interaction with the wild type and Omicron RBDs using all‐atom molecular dynamics (MD) simulations including explicit water. As shown in Figure 3c, LPGS binds to the wild type RBD on the cationic patch away from the RBM, whereas it binds to the cationic patch on the RBM of the Omicron RBD. The per‐residue contact plot highlights the key cationic/polar residues of the wild type and Omicron RBD with which the sulfate groups of LPGS primarily interact (Figure 3d). Although the position of the key residues of RBD interacting with LPGS are different for the wild type and Omicron, the total number of contacts LPGS forms with each of these RBDs is almost the same (Figure 3d inset) and thus the interaction energies of LPGS binding to the two different cationic patches (Figure 3c) agree within the error (see Figure 3e). Since the cationic patch present on the wild type is not modified much in the Omicron variant, LPGS can bind to two cationic patches on the Omicron RBD. Therefore, the binding affinity of polysulfates to the Omicron RBD will be larger compared to the wild type RBD and will exhibit multivalent enhancement for a sufficiently long polysulfate chain which can access both cationic patches. In addition, binding of a polysulfate chain to the new site of Omicron may interfere with interaction between RBD and ACE2.

### Putative Consequences of Spike Protein Mutations of Omicron for Subsequent Steps of Cell Entry

So far, based on literature data and our own experiences we have considered only the very first step of binding to the host cell. Of course, of similar relevance are the mutations in the spike protein for the next steps of cell entry, i. e., binding to the ACE2 receptor and cleavage of the two subunits S1 and S2 by furin (see Figure 2). At this stage we can provide at best hypotheses taking lessons from the former successful variants, such as Alpha (B.1.1.7), Beta (B.1.351; +1), Gamma (P.1; +1) and Delta (B.1.617.2; +4). Several studies have shown that mutations in the RBD, in particular mutations in the RBM such as K417, S477, T478, E484, and N501 also seen in the Omicron variant may enhance binding to ACE2.^[11]^ Thus, this may implicate that even the second step of cell entry, the binding to ACE2 is favored for the Omicron variant. Positively charged amino acids in the RBD/RBM, which are now even more abundant in the Omicron variant, have been reported to favor the ′up′ conformation.^[12]^ Further studies are necessary, if a tighter binding to HSPG by the mechanism discussed above may even increase the binding probability to ACE2. Notably, interaction between the RBM and ACE2 is essentially determined by a network of hydrogen bonds. Recent simulations implicate that mutations in the RBD of the Omicron variant may also engage in hydrogen bonding between the RBD and ACE2.^[13]^ Indeed, although recent experimental studies have found that the affinity of the isolated Omicron RBD to human ACE2 is about two fold with respect to wt,^[14]^ the affinity of the intact trimer spike protein is almost 10 fold higher presumably due to a stabilization of the ‘up’ conformation of the RBD.^[14b]^ A reason for that could be that mutation D614G which has been found in all successful variants including Omicron has been shown to support the ‘up’ conformation of the RBD and thus binding to ACE2.^[15]^

Another indication for an enhanced infection potential of Omicron is given by the mutation P681H which is next to the ‐RRAR‐ furin cleavage site. The enzyme furin of the host organism cleaves the spike protein into the subunits S1 and S2 priming the protein for triggering fusion of the virus envelope membrane with the respective target membrane of the host cell, either the plasma membrane or endosomal membrane, to release eventually the viral genome into the cytoplasm. The very similar mutation P681R in the Delta variant has been shown to facilitate furin mediated cleavage of the spike protein.^[16]^ Finally, also here experimental studies are necessary to characterize the consequences of the mutation P681H for the infection potential of the Omicron variant.

### Perspective

The above overview has clearly revealed that the successive mutants of SARS‐CoV‐2 exhibit an increased number of positively charged amino acids exposed on their surface. The associated changes of the Cardin‐Weintraub motif will certainly enlarge the interaction with the HSPG located on the surface of the host cells (cf. Figure 1). Since this interaction is the first and necessary step for cell infection, we hypothesize that the higher transmissibility of the subsequent variants is directly related to occurrence of these Cardin‐Weintraub sequences at the spike protein surface. A recent study showed that Omicron already replicates much better in the upper respiratory tract, i. e., the bronchus, than wild type itself and other known variants of it. Although the study suggested that the cause may be the efficient expression of ACE2 in bronchial tissues, increased affinity for sulfated GAGs may equally play a role.^[17]^ Notably, at the same time, a change of residue charge due to mutation could increase the resistance of the virus to vaccine‐elicited antibodies.[^1a^, ^18^] However, a recent study completely mapping mutations of the RBD that escape antibody recognition has concluded that the impact of specific mutations of the RBD may vary significantly between antibodies whose target is the same region of the RBD.^[19]^

## Methods

The atomic coordinates of the wild type RBD of the SARS‐CoV‐2 spike protein was obtained from the deposited crystal structure (PDB ID: 6M0J). This structure was taken and required residues (highlighted in Figure 3a) were mutated using PyMOL, to build the Delta and Omicron variant RBDs. The structure of a linear poly‐glycerol sulfate (LPGS) undecamer was built using Avogadro software.^[20]^

All MD simulations were performed at least for 900 ns in the NpT ensemble at T=300 K and p=1 bar with periodic boundary condition in *xyz* directions, using GROMACS 2020.1 package.^[21]^ Other details about the model building, force‐field parameters and the simulation protocol used are the same as given in our earlier publication on the SARS‐CoV‐2 inhibition by polysulfates.^[5]^

Electrostatic potential maps of RBDs were calculated using the APBS tool^[22]^ and visualized using VMD.^[23]^ Simulation snapshots were rendered using VMD. Number of contacts: A contact is counted if an atom of LPGS falls within 3.5 Å of any atom of a protein residue. The total number of such contacts averaged over the last 600 ns of simulation data is presented. The average interaction energy and the standard deviation calculated from the last 600 ns of simulation data is given.

## Conflict of interest

The authors declare no conflict of interest.

## Biographical Information

*Chuanxiong Nie completed his PhD at the Institut für Chemie und Biochemie Freie Universität Berlin. He began his postdoctoral research at Freie Universität Berlin in 2021. His research interests focus on nanostructures against SARS‐CoV‐2*.

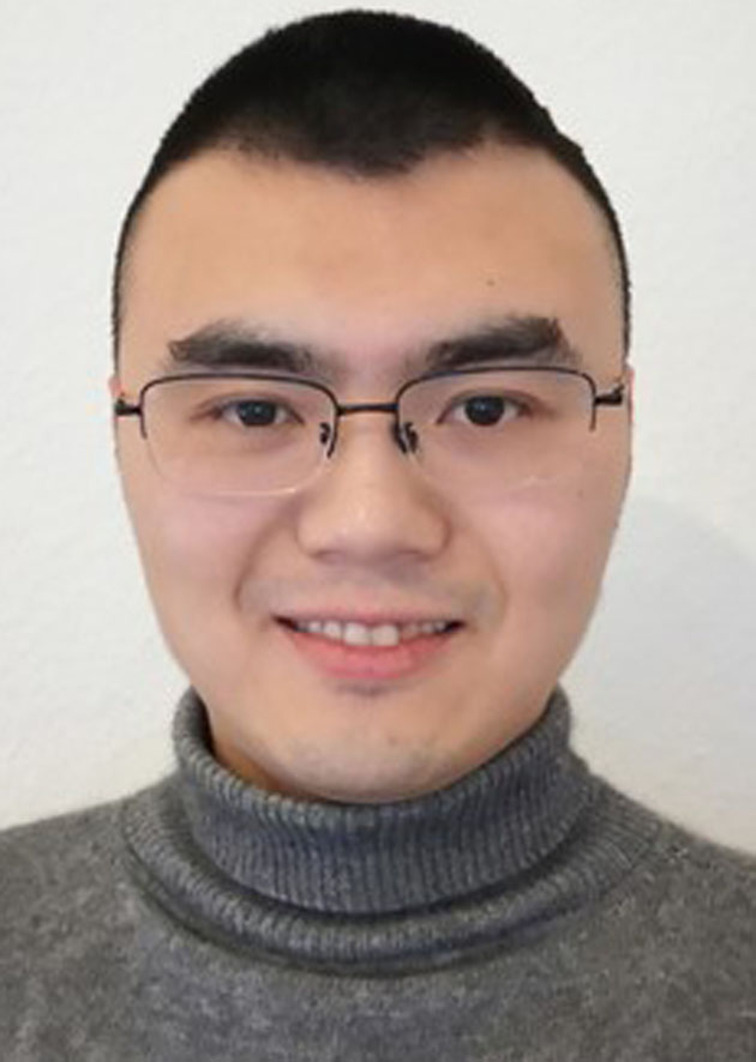

## Biographical Information

*Anil Kumar Sahoo studied physics at the Indian Institute of Science, Bangalore, where he obtained his PhD in computational bio‐ and soft‐matter physics in 2020. He has been working as a postdoctoral researcher at the Freie Universität Berlin and the Max Planck Institute of Colloids and Interfaces, Potsdam, since March 2020, with a focus on hydration effects in biological matter*.

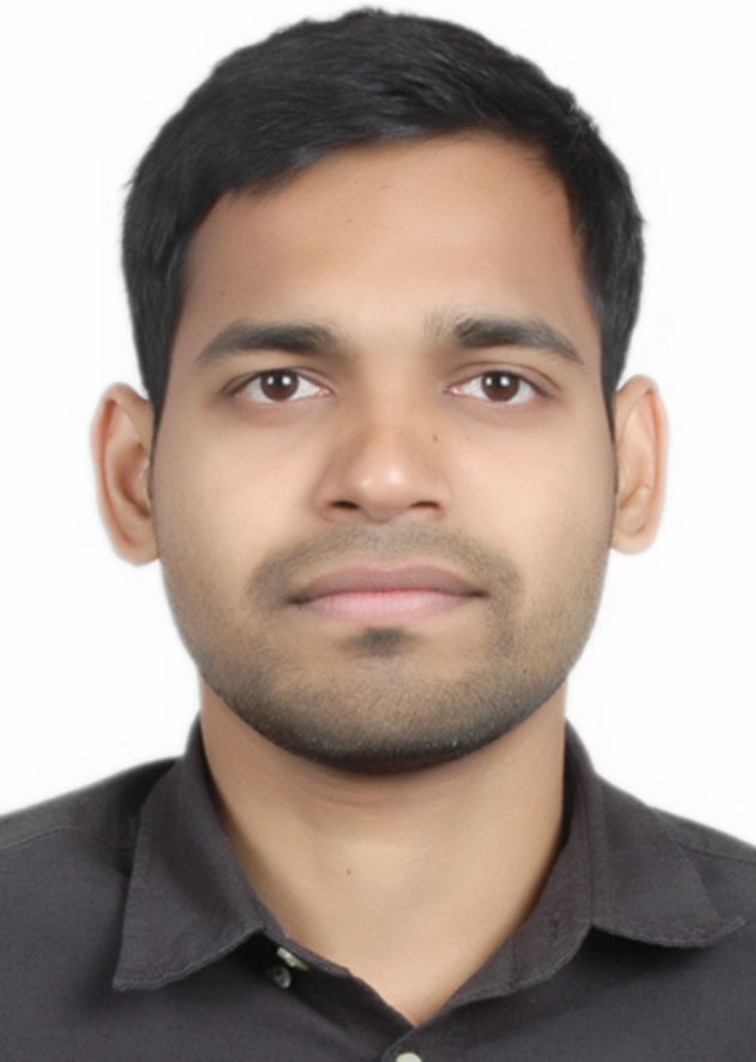

## Biographical Information

*Roland Netz studied physics at the Technical University of Berlin and at MIT and received his Ph.D. in 1994 from the University of Cologne. After postdoctoral positions at Tel‐Aviv University, UC Santa Barbara, Seattle, Institute Charles Sadron in Strasbourg, CEA in Paris, and the Max‐Planck Institute for Colloids and Interfaces in Potsdam, he was appointed associate professor of physics at the LMU Munich in 2002 and full professor of physics at the TU Munich in 2004. Since 2011 he has held a chair in theoretical biosoft‐matter physics at the Freie Universität Berlin. His research focuses on the structure and dynamics of water, proteins, polymers, and charged systems using quantum, atomistic, and coarse‐grained simulations as well as theoretical approaches*.

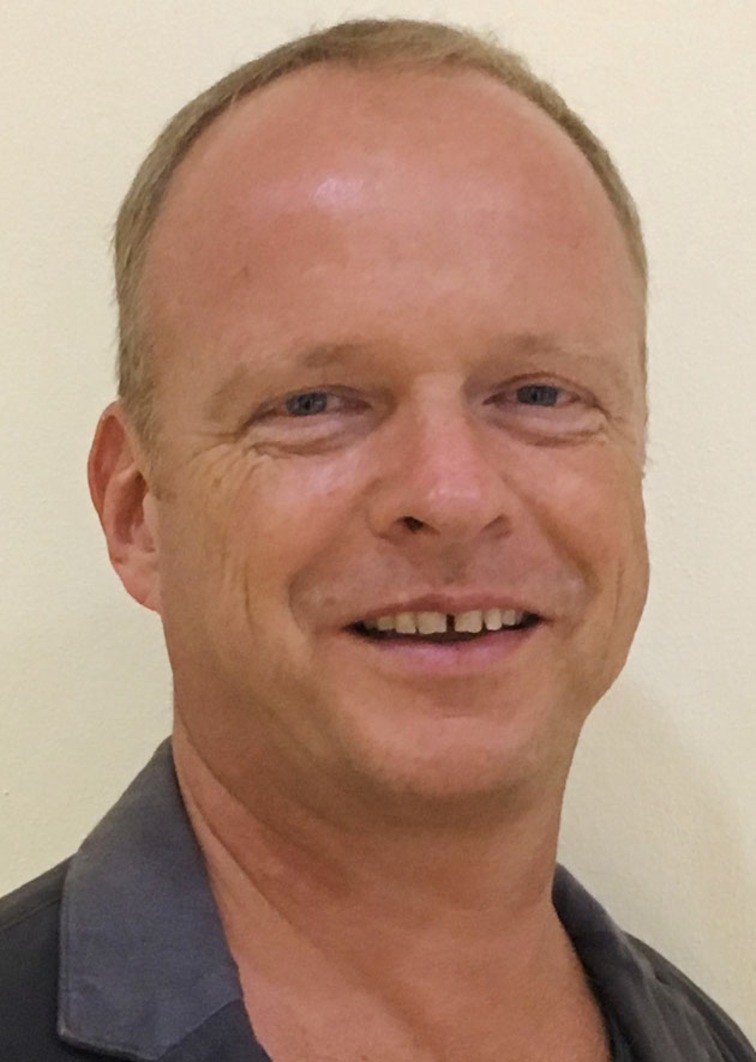

## Biographical Information

*Andreas Herrmann has received his PhD in Biophysics from the Humboldt‐Universität Berlin. He was appointed full professor of Molecular Biophysics in 1993 at the Humboldt‐Universität Berlin and since October 2021 he has been a Senior professor at the Freie Universität Berlin. His main fields of interest cover membrane biophysics with focus on lateral and transbilayer lipid dynamics and on membrane fusion. His current research is focused on molecular mechanisms of cell entry and assembly of enveloped viruses at single virus and cell levels. This includes the development and application of highly efficient biomimetic multivalent inhibitors of virus binding to host cells*.

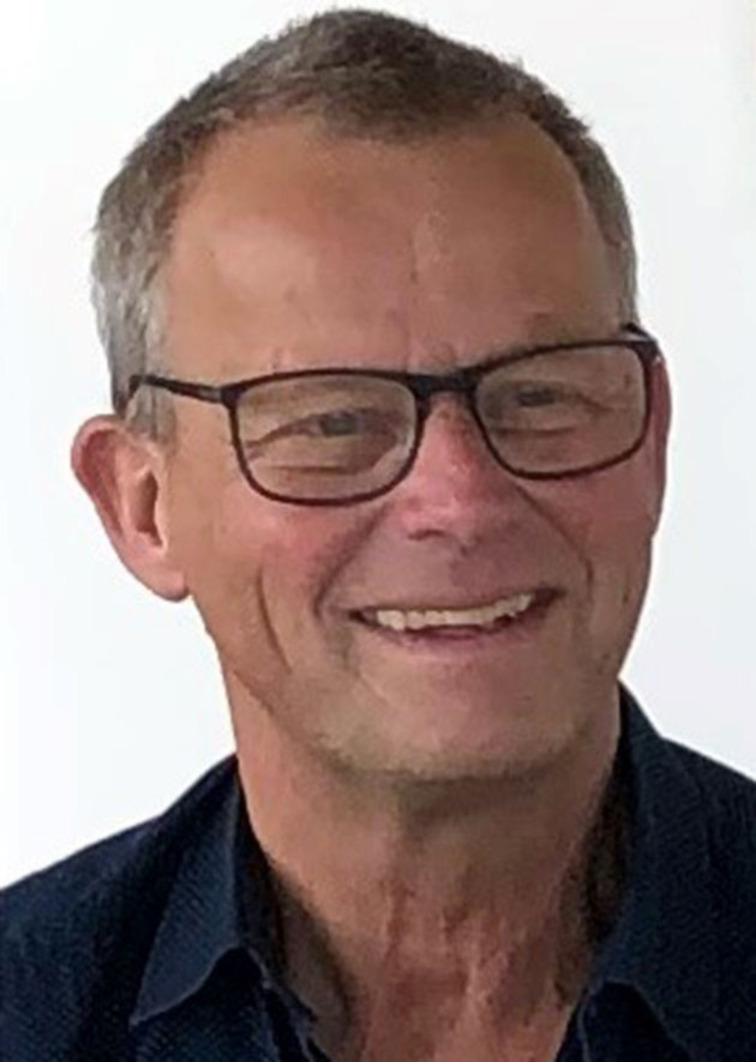

## Biographical Information

*Matthias Ballauff studied chemistry at the University of Mainz where he obtained his PhD in physical chemistry in 1981. After postdoctoral studies with P. J. Flory at Stanford University he joined the Max‐Planck‐Institut in Mainz as a research associate. He was a professor at the University of Karlsruhe (1990–2003) and worked at the University of Bayreuth (2003–2009). In 2009, he joined the Helmholtz‐Zentrum Berlin and became a professor of experimental physics at the Humboldt University Berlin. After his retirement in 2019 he became a guest professor at the Institute of Chemistry and Biochemistry of the Freie Universität Berlin. His research is focused on the structure and interaction of polyelectrolytes*.

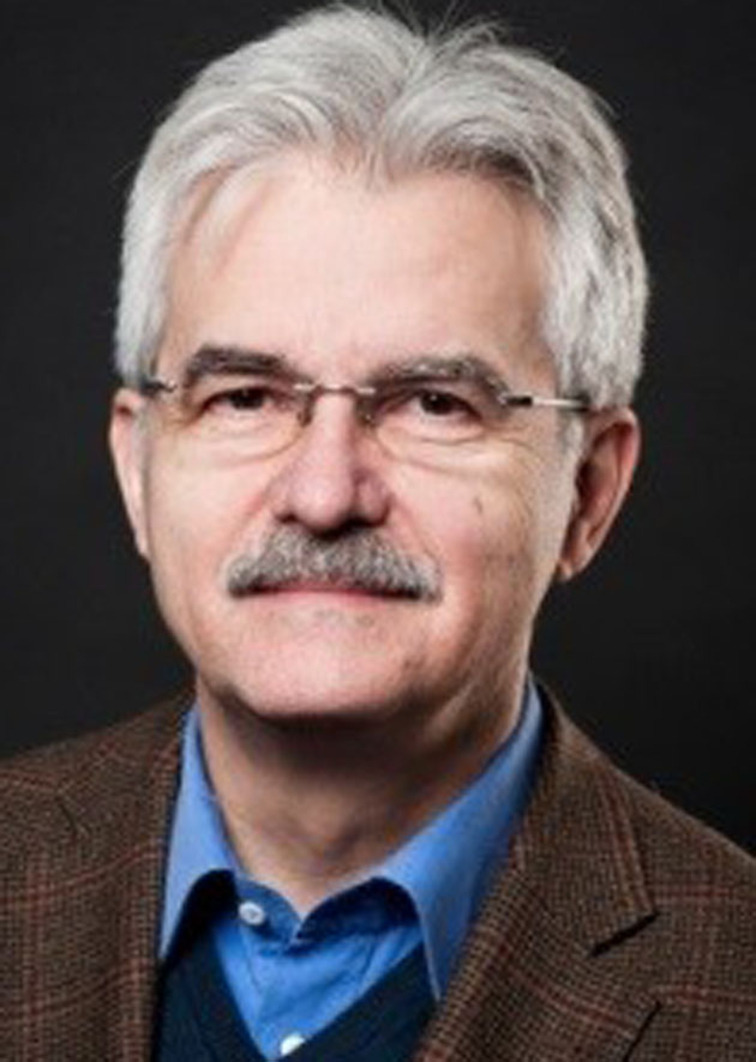

## Biographical Information

*Rainer Haag is Professor of Organic and Macromolecular Chemistry at Freie Universität Berlin. Since 2008 he has been spokesperson for SFB 765 “Multivalency as a Chemical Organization and Action Principle”, and in 2021 he became spokesperson for the SFB 1449 “Dynamics in Hydrogels at Biointerfaces”. His research interests include dendritic and linear polyglycerols as multivalent virus inhibitors, macromolecular nanotransporters for DNA and drug delivery. In 2004, his group received the NanoFutur researcher award of the Federal Ministry of Science and Research. Together with the start‐up company Dendropharm, he received the Berlin‐Brandenburg Innovation Award 2016. Since 2019 he has been an elected member of the German Academy of Science and Engineering (acatech)*.